# Supplementary material for: Knowledge and remaining gaps on the role of animal and human movements in the poultry production and trade networks in the global spread of avian influenza viruses – A scoping review
Source: PLoS One. 2020 Mar 20;15(3):e0230567. doi: 10.1371/journal.pone.0230567 (PMC7083317; doi:10.1371/journal.pone.0230567)
Supplement: S2 Table — Application on Scopus database on the 31 May 2019. (PDF) [file pone.0230567.s002.pdf]

S2 Table. List of the different strings included in the search strategy and the number of retrieved references for each string. Application on Scopus database on the 31 May 2019.

String	Number of papers retrieved
TITLE-ABS-KEY (diffusion OR transmission OR spread OR emergence OR introduction OR outbreak OR epidemiology OR risk OR model)	17 857 593
TITLE-ABS-KEY (influenza)	92 627
TITLE-ABS-KEY (avian OR poultry OR duck OR chicken OR chicks OR geese OR turkey OR quail OR partridge)	523 979
TITLE-ABS-KEY (network* OR organization* OR value-chain OR compan* OR production* OR farm* OR industr* OR sector)	10 464 926
TITLE-ABS-KEY (commercial OR trade OR traffic OR mobility OR movement OR domestic)	3 403 383
Combined search request	
(TITLE-ABS-KEY (diffusion OR transmission OR spread OR emergence OR introduction OR outbreak OR epidemiology OR risk OR model) AND TITLE-ABS-KEY (influenza) AND TITLE-ABS-KEY (avian OR poultry OR duck OR chicken OR chicks OR geese OR turkey OR quail OR partridge) AND TITLE-ABS-KEY (network* OR organization* OR value-chain OR compan* OR production* OR farm* OR industr* OR sector) AND TITLE-ABS-KEY (commercial OR trade OR traffic OR mobility OR movement OR domestic))	1 044
