# Supplementary material for: Knowledge and remaining gaps on the role of animal and human movements in the poultry production and trade networks in the global spread of avian influenza viruses – A scoping review
Source: PLoS One. 2020 Mar 20;15(3):e0230567. doi: 10.1371/journal.pone.0230567 (PMC7083317; doi:10.1371/journal.pone.0230567)
Supplement: S3 Table — UK: United Kingdom, USA: United States of America. (PDF) [file pone.0230567.s003.pdf]

S3 Table. Listing of the objectives, location, type, methods and main results of the 57 selected references. UK: United Kingdom, USA: United States of America

Classification	Type of network studied	Type of study	Objectives of the study	Location	Method to collect data	Method to analyse data	Main results	Reference
Studies which aimed to demonstrate the link between animal, human, fomite movements and AI spread within the poultry production and trade networks	International trade network	Modelling	To determine if the international spread of HPAI H5N1 was influenced by the poultry trade from infected countries	Asia	Official data	Generalized Estimating Equation model	The risk of poultry infection increase in the importing country with the number of live chickens imported.	(30)
		Risk assessment	To evaluate the risk of introduction and dissemination of H5N1 through trade	Ethiopia	Not specified	Qualitative risk assessment model	The risk of HPAI introduction through day old chicks' introduction is negligible.	(29)
		Risk assessment	To estimate the probability of HPAI introduction into Spain through legal trade	Spain	Official data	Quantitative risk assessment model	The risk of HPAI introduction is higher with imports of ducks than chickens or turkeys.	(31)
		Risk assessment	To better understand illegal trade to propose preventive and control options	Vietnam	Questionnaire	Descriptive analysis and qualitative risk assessment	The illegal trade of live poultry represent a high risk of HPAI introduction. This risk of direct exposure is higher for the introduction of illegal batch of spent hens and duckling than batch of day old chicks.	(27)
	National or local trade network	Modelling and network analysis	To assess the network for surveillance and control protocols and biosecurity practices	Vietnam	Cross-sectional survey	Individual based model and network analysis	The live birds markets with the highest connectivity (hubs) always combined high susceptibility (probability to be contaminated) and infectiousness (number of other markets contaminated after the infection was seeded in the market considered).	(33)
		Modelling	To determine the impact of control strategies at live bird markets level on avian influenza spread and to assess the aspects of the system to improve control strategies	China	Scientific literature	meta-population model	Control measure on trade movements such as market closure and rest days have an impact on the expected number of secondary cases (market or farm) per single infected case.	(34)

		Modelling	To assess the network for surveillance and control protocols	Indonesia	Experts interviews and scientific literature	Stochastic simulation model	The probability of infection of a live bird market after movement of live birds from infected village is high.	(35)
		Network analysis	To improve knowledge on live bird trade and to assess the network for surveillance and control protocols	Pacific islands	Cross-sectional survey	Network analysis	Association between the live poultry movement (on and off farm) and the presence of a disease during the last 12 months.	(36)
		Network analysis	To investigate associations between poultry trade characteristics and HPAI H5N1 infection	China	Cross-sectional survey	Network analysis	No significant association between live poultry trade network parameters and HPAI H5N1 infection status of live birds markets.	(37)
		Network analysis	To investigate seasonal patterns in the association between the movement of live poultry and HPAI H5N1 infection	China	Longitudinal survey	Network analysis	Increase of poultry trade is associated with higher risk of HPAI H5N1 infection in human and poultry.	(38)
		Descriptive and network analysis	To improve knowledge on poultry trade network in Vietnam and to analyse it's potential role in the spread of H5N1 HPAI	Vietnam	Cross-sectional survey	Network analysis	Live poultry traders trend to link region with the same H5N1 HPAI infection status.	(39)
		Network analysis	To identify the role of live birds markets biosecurity indicators and poultry movement in H7N9 affected areas	China	Cross sectional survey	Network analysis	The presence of H7N9 AIV in live birds markets is significantly associated with live birds markets that sold chicken to other live birds markets	(40)
	National or local production network	Modelling	To assess surveillance and control protocols	UK	Cross-sectional survey	Stochastic simulation model with a network approach	Modelled outbreak size in duck production was impact by slaughterhouse movements and company workers (sensitive analysis).	(41)
		Modelling	To identify the role of commercial farms in the persistence and the spread of AIVs	UK	Official data	Stochastic simulation model and descriptive analysis	Even if the event is rare, a large spread of HPAI within poultry network is possible. Slaughterhouse-linked movements have an impact on outbreak size and the frequency of company workers movements have an impact on the spread beyond index premises (sensitive	(42)

							analysis). Catching team company movements have little effect on the probability of a large outbreak occurring.	
		Modelling	To identify the role of commercial farms in the persistence and the spread of AIVs / To assess biosecurity practices	USA	Survey	Stochastic simulation model	Off-farm spread most frequently associated with feed trucks and company workers or hired help.	(43)
		Modelling	To quantify the effect of several factors on the spatial distribution of outbreaks in the commercial poultry sector and to generate predictive risk maps for HPAI	France	Official data	Boosted regression tree model	The proportion of infected holdings increased with the density of outgoing movements of fattening ducks.	(44)
		Modelling	To identify the additional risk that a mitigation measure (compartmentalisation) poses	Netherlands	Official data	Generic HPAI transmission risks model	Farms included in the same production company would have a high risk of HPAI transmission.	(45)
		Modelling	To build an epidemiological model to lead an economic analysis of HPAI control	Netherlands	Official data	InterSpread Plus simulation model	The epidemiological indicators are sensitive to the probabilities of transmission for the movement contact. Animal movements considered: poultry, ready to lay parents and parents and grandparents stock; Fomite movements considered: Trucks that have transported manure, the collection of consumption and hatching eggs;<br>Human movements considered : loading/unloading team, veterinarian.	(46)
		Modelling	To assess the impact of various control measure (compartmentalisation vs zoning)	UK	Official data	Metapopulation model and SIR model	Reduction of the AI spread with control measures on these movements.	(47)
		Modelling	To identify the role of commercial farms in the persistence and the spread of AIVs	UK	Official data and private data (catching companies)	Deterministic compartmental model (SEIR)	A large fraction of farms (28%) have a high relative transmission of AIV risk and a high between farms association.	(48)

		Modelling	To identify the role of commercial farms in the persistence and the spread of AIVs	USA	Official data and scientific literature and expert interviews	NAADSM model	Indirect contacts (e.g. workers, vaccination team, cleaning disinfection team, feed deliveries) would have a more important role in the between-farm transmission than direct live birds contact	(49)
		Modelling	To assess surveillance and control protocols and consequences of AIV introduction	UK	Official data	Stochastic model	Possibility of a wide spread of HPAI infection within production network (even if the event is rare) according to a model which take into account 4 ways of transmission: transportation to slaughterhouses, feed deliveries, company transmission and local transmission	(50)
		Modelling and network analysis	To evaluate control strategies in the case of AIV introduction	UK	Official data and survey	Group structure model and fixed network model	Impact of control measures on several routes of transmission (e.g. slaughterhouses, vehicles and personnel from catching companies and within production company) on HPAI spread	(51)
		Network analysis	To identify the role of commercial farms in the persistence and the spread of AIVs	Korea	Survey	Social network analysis	The degree centrality analysis showed that the centrality of infected farms in poultry business networks (medicine and feed) is higher than for non-infected farms	(52)
		Network analysis	To develop and assess surveillance and control protocols	South Africa	Official data	Network analysis (time series analysis and comparative analysis)	Infected farms are more central and more connected than the rest of the network	(53)
		Risk assessment	To identify the role of commercial farms in the persistence and the spread of AIVs and risk factors	USA	Survey	Quantitative risk assessment model	Risk of between farm transmissions is largely driven by company affiliation. Part-time workers employment contributes to significant increase the risk in most risk scenarios.	(54)
		Risk assessment	To identify the role of commercial farms in the persistence and the spread of AIVs/ To identify risk factors of AIV spread	Australia	Expert interviews and scientific literature	Quantitative risk assessment model	The pathways of HPAI and LPAI spread between farms with the highest average median probability are bird pick-up system, egg trays, egg pallet and farm workers	(55)

		Risk assessment	To investigate the pathways of AIV introduction and spread	Australia	Expert interviews	Expert elicitation	Shared bird pick up transport was considered to be most likely pathway of spread from broiler farms for both LPAI and HPAI while shared egg trays and pallets were considered to be most likely pathways for the layer operations. Dead bird pickups were considered a substantial threat to spread of LPAI and HPAI on broiler farms. Shared bird pick up transport, shared farm workers, and shared equipment were other pathways that were considered to be of risk for HPAI in layers free range.	(56)
Studies which aimed to describe animal, human and fomite movements in a context of AI spread within poultry production and trade networks (but with no direct demonstration of the link)	International trade network	Descriptive	To describe of genetics value-chain	Global	Experts interviews	Descriptive	Description of international trade of hatching eggs and day old chicks	(28)
	National or local trade network	Network analysis AND Modelling	To assess the network for surveillance and control protocols	Vietnam	Cross-sectional survey	Stochastic model and network analysis	Description of trade network AND Within live bird markets epidemiological model	(32)
		Network analysis	To describe live poultry movement through Cambodia to understand how these movements could influenza HPAI spread/ To assess the network for surveillance and control protocols	Cambodia	Cross sectional survey	Network analysis	Description of poultry trade and live birds markets networks including mention of international trade of live birds with neighbouring countries	(61)
			To develop and assess surveillance and control protocols	Vietnam	Experts interviews and focus group discussion	Value-chain analysis	Description of free-grazing ducks trade value-chain	(58)
			To improve knowledge on poultry trade and To assess the network for surveillance and control protocols	Mali	Cross-sectional survey	Network analysis	Description of poultry trade network	(59)

			To assess the network for surveillance and control protocols	Bangladesh	Cross-sectional survey	Network analysis	Description of poultry trade network	(60)
		Descriptive/network analysis	To describe the informal poultry trade in the aim to improve control and prevent disease spread	Kenya	Cross-sectional survey	Network analysis and statistical analysis	Description of poultry traders movements	(57)
		Descriptive analysis	To improve knowledge on live bird markets and birds' movements	China	GPS-GSM data	Analyse of directional data	Description of the trade network of live ducks	(62)
			to quantify the traceability of poultry from farms to live bird markets	Vietnam	Questionnaire and tag of batches	Descriptive statistics, univariable analysis and multivariable analysis	Description of poultry traders movements	(63)
	National or local production network	Network analysis	To assess biosecurity practices	Indonesia	Cross-sectional survey	Value-chain analysis	Description of duck and broiler value chain including day-old chicks movements	(64)
			To develop and assess surveillance and control protocols, To describe the poultry production network To assess the potential impact of introduction	Kenya	Official data	Value-chain analysis	Description of poultry production network based on animal movements	(65)
			To assess surveillance and control protocols	Egypt	Survey	Social network analysis	Description of poultry production network with a focus on day-old chicks movements	(66)
			To analyse the value chain to distinct the effect of the disease on the different stakeholders and to localise the critical control points where the disease can enter the value-chain	Nigeria	Semi structured interviews and focus group discussions	Value chain analysis	Description of poultry value-chain	(67)
			To develop awareness and to develop control strategies	Nepal	Cross sectional survey and focus group	Value chain analysis	Description of poultry value-chain	(68)

			To understand the nature of the value chain and the impact of AIV on the value chain	Indonesia	Scientific literature, cross-sectional survey	Value-chain analysis	Description of poultry value-chain	(69)
			To describe the topology of the contact and movements and to discuss how this description can be used to refine the biosecurity and surveillance strategies	China	Expert opinion elicitation and survey questionnaires	Social network analysis	Description of two poultry value chains	(70)
		Descriptive/Statistical analysis	To assess surveillance and control protocols / to clarify the contact structure within a poultry production network	UK	Official data	Descriptive analysis	Description of animal, fomite and human movements within poultry production network	(71)
			To compare the contacts between commercial and non-commercial farms	Switzerland	Official data and cross-sectional survey with questionnaire	Multinomial model	Description of poultry, fomite and human movements within poultry production network	(72)
			To describe the poultry production network/ To assess the potential impact of HPAI introduction	Australia	Official data	Descriptive analysis	Description of four poultry industries (broilers, layers, turkeys and ducks) including animal, fomites and human movements.	(73)
			To clarify the contract structures within the poultry production network	UK	Survey	Statistical analysis and statistical modelling	Analysis of between-farm associations based on animal, fomites and human movements	(74)
Studies which aimed to describe AI spread within the production and trade networks (without making explicit links to animal, human and	National of local trade network	Network analysis AND Modelling	To assess the network for surveillance and control protocols	Vietnam	Cross-sectional survey	Stochastic model and network analysis	Description of trade network AND Within live bird markets epidemiological model	(32)
		Modelling	To analyse the spread of AIVs among commercial farms and to assess surveillance and control protocols	India	Scientific literature	Deterministic SEIR model	Epidemiological model of AI spread between wild birds, backyards, farms and live birds markets	(75)
	National or local production network	Modelling	To explore the impact of climatic and economic factors on H5N1 HPAI spread seasonality in domestic poultry	Vietnam	Official data	SIRS model	Epidemiological model between farms considering seasonal variation	(76)

fomite movements)		Modelling	To build a spatial transmission model which fit the epidemic spread and to assess control measures	Italy	Official data	SEIR model	Epidemiological model of between farms AI spread	(77)
		Statistical analysis	To analyse the spatio-temporal distribution of outbreaks and to estimate the spread rate	France	Official data	Trend surface analysis	Spread rate analysis of HPAI H5N1	(78)
		Modelling	To develop a model to better understand the spread modalities of AIVs and To assess surveillance and control protocols	not specific	Scientific literature	SIR model and network analysis	Generic epidemiological model of AI spread	(79)
		Modelling	To propose a new approach to represent the spread of avian influenza with boolean network model	USA	Official data and data from the scientific literature	SIRS Boolean model	Epidemiological model of AI spread	(80)
		Modelling	To assess surveillance and control protocols	Netherland	Official data	Combination between stochastic and deterministic model	Epidemiological model of AI spread to assess control strategies	(81)
		Modelling	To analyse the spread of AIVs among commercial farms and to assess surveillance and control protocols	Italy	Survey	Deterministic SEIR model	Within farm epidemiological model of two AI strains spread	(82)
		Modelling	To assess control strategies	Nigeria and Ghana	Survey and scientific literature	gravity model of transmission	Epidemiological model assuming that the probability of AI spread between premises through areal spread, animal movements and fomites is function of centroid distance between premises	(83)
