# Supplementary material for: Knowledge and remaining gaps on the role of animal and human movements in the poultry production and trade networks in the global spread of avian influenza viruses – A scoping review
Source: PLoS One. 2020 Mar 20;15(3):e0230567. doi: 10.1371/journal.pone.0230567 (PMC7083317; doi:10.1371/journal.pone.0230567)
Supplement: Attachment — Submitted filename: suggestions_feedback to the authors.pdf [file pone.0230567.s004.pdf]

Comments and suggestions to the author(s):

In this study, Hautefeuille et al. conducted a scoping review according to the PRISMA guidelines extended for Scoping Reviews, with the primary goal of identifying gaps in the literature concerning the role of animal and human movements within the poultry production and trade networks that contribute to the global spread of avian influenza virus. The authors examined the extent, range and nature of the evidence on this topic, summarizing findings form a body of knowledge encompassing heterogeneous sources. As avian influenza continues posing threats to animal and human health, such a scoping review is of particular interest to researchers and policymakers as it may aid the planning and commissioning of future research.
However, some issues need to be properly addressed and discussed by the authors.

Major concerns

The manuscript needs to be professionally proofread. Incomplete and/or long sentences and concepts, poor grammar or punctuation detract from readability and hamper the scientific merit and originality of this manuscript, so it needs major rewriting for sense and flow. The grammar and English revision is beyond my task, therefore I can only provide few examples along the manuscript that were particularly hard to understand:

Page 1 lines1-2: the title itself to begin with; as it is stated it is not clear that this manuscript is a scoping review aiming at mapping evidence/identifying main concepts, theories, sources, and knowledge gaps on the role of animal and human movements within the poultry production and trade networks in the global spread of AI viruses.

Page1-2 lines 33-40: two sentences repeating the same concepts, but remaining vague on the conclusion. It is clear that there is a general awareness of the role of animal and human movements in the local and global spread of AI, but it is not clear what the authors identified as gap(s) of knowledge: they only mention 'fine understanding' in line 35 and 'limited work has been carried out to study it' on lines 38-40. Do they mean that no study quantified the proportion of AI virus transmission/spread due to animal and/or human movements within poultry production and trade networks? Do they mean that no study identified which is the most important route(s) of transmission/spread of AI within the production and trade networks? It would be worthy to elaborate a more precise, though concise sentence, on this issue in the abstract and then to elaborate on the concept throughout the manuscript, when commenting the groups of studies selected. For example on page 11 lines 222-224 the sentence is not clear enough. What the authors refer to when they say 'this study'? Which of the studies mentioned in the paragraph lines 215-224.

Page 7 lines 135-137: 'on title, abstract and full text if necessary using the following exclusion criteria on title, abstract and full text if necessary', two repetitions in one sentence.

Page 9 lines 180-182: this sentence is not clear, and it seems incomplete. Please rephrase.

Page 9 line 192: in the figure 2 title, it says 'number without comma', but there is no number with or without comma in the figure itself. Please clarify.

Page 8 line 167; page 11 line 211: Spell out numbers (e.g., one, two, three) at the start of sentences rather than using numerals.

Page 10 line 205: in the title of Table 1, please explain the colour code used in the table.

Moreover, it would help to clarify the following concepts and passages in the text:

In the Introduction, please be more specific with respect to the years considered in the review: explain the reason why AI outbreaks were considered only starting from 2010 (page 3 lines 45; line 55; line 61), although the search strategy included years from 1975 to 2019 (page 5 line 104-105).

Material and methods: it would help to find the same section structure as provided in the checklist/S1 Table and that is: protocol & registration; eligibility criteria; information sources ect… Those information are all present in the text but not is a structured manner, which is also one of the principles of the PRISMA evidence-based system for reporting items in reviews.

Results: unclear, but key components are present and major rewriting can address lack of clarity

Page 11 lines 209 onwards: In the results it would help to find the same structure outlined in Figure 1 (Flow chart diagram), so that the details concerning the references could be discussed in three different sections reflecting the three groups/boxes printed in Fig. 1. As it is discussed now, it is not clear enough.

Figure 1: please be more specific in the definition assigned to the three final groups of references selected, which do not correspond to what is reported in the text (page 11 lines 216-220)
-First box, 28 references on the role of animal and human movements […]: what do you mean by 'the role'? At page 11 line 217, those 28 references are referred to as corresponding to references on the link between animal and human movements, which are the references in the second box, as reported in Figure 1;
-Second box, 20 animal and human movements in link with […]: what does it mean 'in link with AI spread'? The entire review is about literature on animal and human movements identified within the poultry production and trade network that are linked to AI spread.
-Third box, 10 references on AI spread within poultry production and trade network […]: are those references specifically quantifying the circulation of AI viruses only within these environments and transmission/spreading mechanisms specifically ascribed to animal and human movements?

Discussion: it lacks of structure. The Discussion should start with one or two sentences that prepare the ground for making considerations on the results obtained. Instead, the authors, after the first sentence about the structured methodology used, started with listing the major limitations or risks and how they were dealt with. Gaps in knowledge are stated only on page 26 lines 399-400.

Page 28 lines 460-463: one reference on risk assessment conducted during an endemic period against many other risk assessment studies conducted in non epidemic periods, does not seem to be a solid ground on which drawing such a conclusion. Please elaborate more on this idea or provide more details on the references and their perspective.

Conclusions: page 30 lines 497-501, repetitions of concepts. Please make one sentence out of the concept repeated concerning the fact that the role of animal and human movements within poultry production and trade networks is acknowledged but poorly studied. Moreover, please elaborate on which aspect(s) is/are poorly studied and can be topic for future research.
